# Non-Hodgkin Lymphoma Manifesting as Bilateral Tonsillar Hypertrophy: A Case Report

**DOI:** 10.7759/cureus.78628

**Published:** 2025-02-06

**Authors:** Hashim S Almishhadany, Asal A Al Azzawi

**Affiliations:** 1 General Practice, Emirates Health Services, Sharjah, ARE; 2 General Practice, University of Sharjah, Sharjah, ARE

**Keywords:** lymphoma, non-hodgkin, sore throat, tonsillar hypertrophy, tonsillar tumor

## Abstract

Non-Hodgkin lymphoma (NHL) is a diverse group of lymphoid malignancies, with varied clinical presentations depending on the anatomical site of involvement. Although typically presenting with nodal disease, extranodal manifestations are not uncommon. NHL involving Waldeyer's ring is considered rare, with the palatine tonsils being the most frequently affected location. This case report presents a 30-year-old male patient with bilateral tonsillar hypertrophy, ultimately diagnosed as NHL. The patient presented with a persistent sore throat, dysphagia, and snoring associated with sleep disturbances. Detailed physical examination, imaging studies, and histopathological analysis of the excised tonsillar tissue confirmed the diagnosis. This case underscores the importance of considering NHL in the differential diagnosis of persistent tonsillar enlargement to ensure timely and appropriate management.

## Introduction

Lymphomas are a heterogeneous group of hematologic malignancies originating from lymphoid tissues, characterized by the clonal proliferation of malignant lymphocytes within the lymphatic system. They are broadly classified into Hodgkin lymphoma (HL) and non-Hodgkin lymphoma (NHL), distinguished by their histopathological and molecular characteristics. NHL encompasses a heterogeneous group of hematologic malignancies originating from B-cells, T-cells, or natural killer (NK) cells. NHL is the most common type of lymphoma, representing approximately 90% of all lymphoma cases and compromising nearly 3% of all cancer diagnoses, making it the most commonly diagnosed hematologic malignancy worldwide [1,2]. However, only approximately 10% of patients with NHL present with extranodal disease in the head and neck region. Furthermore, more than half of these head and neck lymphomas occur in Waldeyer's ring, and 40%-50% of these arise from the tonsil [3]. Non-Hodgkin lymphoma involving Waldeyer's ring (comprising the tonsils, nasopharynx, and base of the tongue) is considered rare, with the palatine tonsils being the most frequently affected location [4,5].

The clinical presentation of NHL is highly variable and depends on the anatomical site of involvement and the specific subtype of lymphoma [6]. Common symptoms include lymphadenopathy, fever, night sweats, and weight loss [6]. When NHL involves the tonsils, patients may present with symptoms that mimic benign conditions such as chronic tonsillitis, making diagnosis challenging. Early recognition through appropriate diagnostic strategies, including imaging and biopsy, along with timely therapeutic interventions such as chemotherapy, is crucial for optimal patient outcomes.

This case report describes a patient with bilateral tonsillar hypertrophy secondary to NHL. The patient's initial presentation of persistent sore throat and dysphagia, combined with the physical examination findings, warranted further investigation [3,7]. The subsequent histopathological examination of the tonsillar tissue revealed NHL, highlighting the necessity of considering this malignancy in cases of unexplained tonsillar enlargement [3].

## Case presentation

A 30-year-old male patient presented to the otolaryngology clinic with a sore throat of moderate intensity that had persisted for the past two months. The patient reported increased snoring and sleep disturbances but denied any associated fever, night sweats, or significant weight loss. He experienced dysphagia, particularly with solid foods, but did not report any other symptoms. The patient's medical history was unremarkable, with no prior surgeries or significant family history of malignancies.

**Figure 1:**
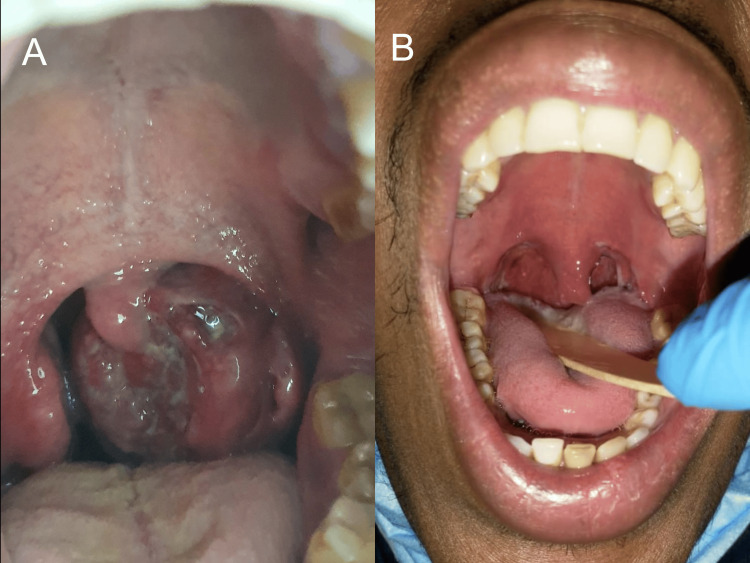
Physical examination of the tonsils (A) Physical examination of the throat upon presentation, before tonsillectomy. (B) Physical examination of the throat after tonsillectomy.

On physical examination (Figure 1), the patient exhibited bilateral tonsillar hypertrophy, with the left tonsil being more enlarged than the right. The left tonsil displayed ulceration and crossed the midline, pushing the uvula. The size of the left tonsil was approximately 5 cm, and the left side appeared cyanotic. No palpable lymph nodes were detected in the neck region.

Laboratory investigations included a complete blood count (CBC) and other relevant tests. The results are highlighted in Table 1.

**Table 1:** Laboratory results and blood film WBC: white blood cell, RBC: red blood cell, Hb: hemoglobin, HCT: hematocrit, MCV: mean corpuscular volume, MCH: mean corpuscular hemoglobin, MCHC: mean corpuscular hemoglobin concentration, RDW: red blood cell distribution width, MPV: mean platelet volume, ESR: erythrocyte sedimentation rate, CRP: C-reactive protein

Test	Result	Reference
WBC	10.3 × 10^9^/L	4-11 × 10^9^/L
RBC	5.53 × 10^12^/L	4.7-6.1 × 10^12^/L (males)
Hb	15.9 g/dL	13.5-17.5 g/dL (males)
HCT	46.30%	40%-50% (males)
MCV	83.7 fL	80-100 fL
MCH	28.8 pg	27-33 pg
MCHC	34.4 g/dL	32-36 g/dL
RDW	12.30%	11.5%-14.5%
MPV	8.5 fL	7.4-10.4 fL
Neutrophil absolute	6.8 × 10^9^/L	2-7.5 × 10^9^/L
Lymphocyte absolute	2.6 × 10^9^/L	1-3.5 × 10^9^/L
Monocytes absolute	0.7 × 10^9^/L	0.2-0.8 × 10^9^/L
Eosinophils absolute	0.1 × 10^9^/L	0.02-0.5 × 10^9^/L
Basophils absolute	0.1 × 10^9^/L	0-0.1 × 10^9^/L
Neutrophil %	66.30%	40%-70%
Lymphocyte %	24.90%	20%-40%
Monocyte %	7%	2%-10%
Eosinophils %	1.20%	1%-6%
Basophils %	0.60%	0%-2%
ESR	5 mm/hour	0-15 mm/hour (males)
CRP	5.9 mg/L	<5 mg/L
Blood group	O+	N/A
Blood film	Normocytic normochromic RBCs, normal WBC count and differential, reactive changes in neutrophils, vacuolations in monocytes, and adequate platelets	

Preoperative imaging

**Figure 2:**
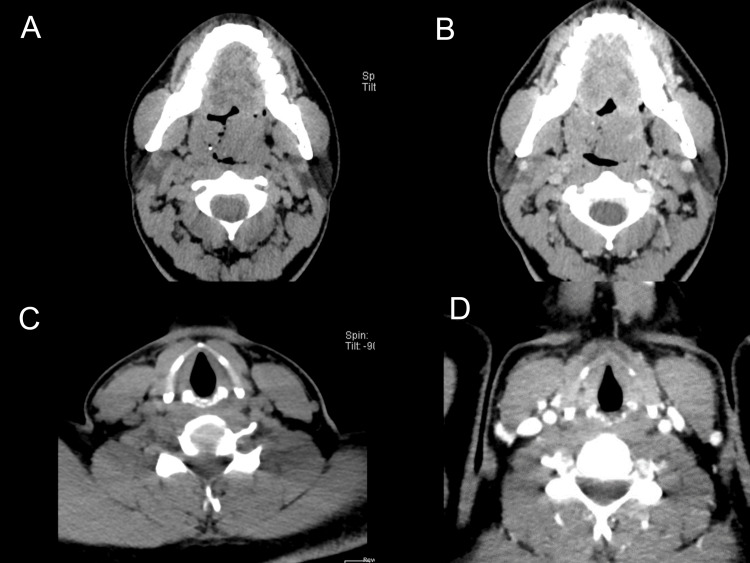
CT scan of the oropharynx (A and B) and the neck (C and D) (A) CT scan of the oropharynx. (B) CT scan of the oropharynx with contrast. (C) CT scan of the neck. (D) CT scan of the neck with contrast. CT: computed tomography

Preoperative imaging included contrast-enhanced CT scans of the neck and oropharynx (Figure 2). Based on the CT scan of the oropharynx, both faucial tonsils were significantly enlarged, more so on the left side. The tonsils exhibited isodense soft tissue attenuation with well-defined outlines, lobulated margins, and significant enhancement, but no cystic components or breakdown. Punctate foci of high attenuation suggested calcifications, more numerous on the left side. The enlargement caused significant narrowing of the oropharynx and displacement of the epiglottis posteriorly, compromising the pre-styloid compartments of the parapharyngeal spaces. The right gland measured 41 × 29 × 35 mm and the left gland 36 × 25 × 54 mm. Both jugulodigastric lymph nodes showed similar attenuation and enhancement patterns to the tonsils, with significant enlargement. Multiple subcentimetric lymph nodes were noted in the jugular groups and left submandibular glands. The nasopharynx, carotid sheaths, masticator spaces, parotid and submandibular glands, laryngeal and hypopharyngeal structures, and retropharyngeal, perivertebral, and posterior cervical spaces, as well as the thyroid gland, all appeared normal. The findings suggested a bilateral marked enlargement of both faucial tonsils, likely due to a granulomatous or chronic inflammatory process, although lymphomatous infiltration could not be excluded and required histopathological assessment.

Based on the CT scan of the neck, the patient presented with numerous bilateral subcentimetric enhancing lymph nodes in the jugulodigastric, jugular, and left submandibular regions, without evidence of infiltration, cystic components, or calcifications. The nasopharynx and oropharynx showed normal patency and configuration. The parapharyngeal, masticator, pterygopalatine, and pterygomaxillary spaces appeared normal, as did the parotid and submandibular glands. The laryngeal and hypopharyngeal structures were intact, with no retropharyngeal pathology. The perivertebral spaces and posterior cervical spaces were unremarkable, and the thyroid gland was of normal size and homogeneous enhancement. The impression indicated multiple residual subcentimetric cervical lymph nodes, suggesting a regressive course of the disease process.

Tonsillectomy and biopsy

The patient underwent bilateral tonsillectomy under general anesthesia. Intraoperative findings included significant hypertrophy of both tonsils, with the left tonsil being larger and crossing the midline. The tonsils were excised using a cold steel dissection technique, and hemostasis was achieved with electrocautery. The excised tissues were sent for histopathological analysis.

Histopathological examination of the excised tonsillar tissue showed effacement of the normal tonsillar architecture by a dense infiltrate of atypical lymphoid cells. Immunohistochemical staining revealed the following results highlighted in Table 2.

**Table 2:** Immunohistochemical staining test

Test	Finding
CD20	Positive in follicular and diffuse areas
CD3	Positive in reactive T lymphocytes
BCL2	Positive
BCL6	Positive
CD10	Faint positivity
MUM	Diffuse positive
CD21	Positive in follicular areas, negative in diffuse area
CD23	Negative
CD5	Negative
c-Myc	Scattered positive in 30%-40% of cells
CD30	Negative
Ki-67	60%

Postoperative imaging

Based on the postoperative CT scan of the chest, abdomen, and pelvis with contrast, the posterior basal segment of the left lower lobe revealed a few peripheral subpleural small soft tissue lesions likely representing pulmonary infiltrates, without significant parenchymal distortion or enhancement. A subcentimetric well-defined enhancing lymph node was noted in the aortopulmonary window. Both lungs appeared clear with no other focal or diffuse parenchymal pathological changes, and the mediastinal vascular structures, heart, and pericardial sac were normal. No hilar lymph nodal enlargement, pleural collections, or chest wall lesions were detected. The abdominal scan showed normal liver, gallbladder, pancreas, spleen, kidneys, and suprarenal glands. The gastrointestinal tract appeared well opacified with no abnormal findings. Multiple subcentimetric and enhancing lymph nodes were observed in the superior mesenteric, intermesenteric, and ileocolic regions, as well as bilateral inguinal lymph nodes, without evidence of cystic components or calcifications. The findings suggested residual lymphadenopathy requiring clinical and laboratory correlation.

On ultrasound of the abdomen, there were excessive abdominal gases but otherwise unremarkable findings, with normal liver, gallbladder, kidneys, and spleen and no ascites or identifiable enlarged lymph nodes.

On positron emission tomography (PET)-CT scan of the whole body, findings were consistent with stage 2 NHL. The disease was localized to the tonsils and adjacent cervical lymph nodes, with no evidence of distant nodal or extranodal involvement. The overall disease burden was minimal, with no significant involvement of other organs or systems.

Final diagnosis and treatment

The final diagnosis was diffuse large B-cell lymphoma (DLBCL) (50%) and high-grade follicular lymphoma (50%). The grade of the follicular lymphoma was grade 3B, raising high concerns for potential transformation to DLBCL. The patient was referred to the oncology department for further management, including chemotherapy. The patient underwent treatment with the R-CHOP regimen, which includes rituximab, cyclophosphamide, doxorubicin, vincristine, and prednisone. This standard first-line therapy for NHL was administered to target the malignant lymphoid cells effectively. The patient responded well to treatment, with a significant reduction in tonsillar size and improvement in symptoms over the course of therapy.

Following the completion of the prescribed cycles, follow-up PET scans demonstrated a progressive decrease in metabolic activity, ultimately confirming complete metabolic remission. With no evidence of active disease, the patient was successfully treated and remained asymptomatic during subsequent follow-up evaluations.

## Discussion

Non-Hodgkin lymphoma accounts for a small proportion of oral cancers, with Waldeyer's ring (comprising the tonsils, nasopharynx, and base of the tongue) being the most frequent extranodal site [3,8]. Published studies show that the peak incidence occurs in individuals in their 60s and 70s, with a higher prevalence in men. The most common histological type is diffuse large B-cell lymphoma, which is an aggressive form, although less frequently, T-cell variants can also be involved [9]. The head and neck area is the second most common site of extranodal lymphoma, with the tonsils being the most common site of involvement; other sites include the nasopharynx and tongue base. Diffuse large B-cell lymphoma is the most frequent histological subtype [10].

The identification of specific biological and molecular markers is essential for accurate diagnosis, prognostic assessment, and treatment planning. Elevated levels of β2-M, LDH, CA 125, TNF-α, IL-2, and sCD44 are all associated with increased tumor burden and poor prognosis in NHL. High β2-M and LDH levels indicate increased cell turnover and aggressive disease behavior, while elevated CA 125 reflects tumor load and is used in staging [11]. Increased TNF-α, IL-2, and sCD44 levels are linked to high tumor burden and poor prognostic criteria, suggesting their potential as prognostic markers [12].

In terms of immunohistochemical markers, CD20-negative NHL is associated with extranodal involvement, atypical morphology, aggressive clinical behavior, resistance to standard chemotherapy, and poor prognosis. Similarly, CD5 expression in DLBCL correlates with worse overall survival. C-MYC rearrangements, especially when concurrent with BCL2 and/or BCL6 rearrangements (double-hit or triple-hit lymphomas), are strongly associated with a very poor prognosis. A high Ki-67 proliferation index in DLBCL also indicates reduced overall survival [13-15]. On the other hand, some markers are associated with a better prognosis. BCL6 expression is linked to improved overall survival, and CD10 expression is associated with longer progression-free survival. CD30 positivity has also been linked to a more favorable prognosis. In contrast, MUM1 expression does not show a significant correlation with clinical and pathological parameters or survival [16]. Finally, while CD3, CD21, and CD23 are valuable diagnostic and classification markers in NHL, their direct prognostic significance remains unclear, and further studies are needed to determine their roles in prognosis [17-19]. In regard to our case, results show a BCL2/BCL6-positive, CD10-faint, CD5/CD23-negative phenotype, suggesting a germinal center-derived lymphoma, likely DLBCL or transformed follicular lymphoma. The moderate Ki-67 (60%) and scattered c-Myc positivity indicate a somewhat aggressive clinical course, but the absence of strong MYC positivity and CD5 negativity may suggest a more favorable prognosis than double-hit or highly proliferative lymphomas.

This case highlights the presentation of high-grade follicular and diffuse large B-cell NHL as bilateral tonsillar hypertrophy, underscoring the importance of thorough clinical evaluation and a high index of suspicion in such cases. The patient's history of persistent sore throat, dysphagia, and ulceration suggestive of malignancy on physical examination warranted further investigation, leading to the diagnosis of NHL.

The contrast-enhanced CT scan of the neck provided valuable information about the size and characteristics of the tonsillar masses and the presence of cervical lymphadenopathy. The surgical plan to perform bilateral tonsillectomy was carried out successfully, and biopsy was essential to establish the diagnosis of NHL.

Histopathological examination remains the gold standard for diagnosing NHL. In this case, immunohistochemical staining was essential in identifying the specific subtype of lymphoma, guiding the treatment plan. The presence of both diffuse large B-cell lymphoma and high-grade follicular lymphoma in the tonsillar tissue highlighted the complex nature of NHL where it appears undiagnosed follicular lymphoma has undergone histological transformation into diffuse large B-cell lymphoma in this case. Imaging studies postoperatively played a crucial role in assessing the extent of the disease. The CT scan of the chest and abdomen, coupled with a PET-CT scan on the whole body, helped rule out systemic involvement, while the ultrasound of the abdomen confirmed the absence of abdominal lymphadenopathy.

The management of tonsillar NHL involves a multidisciplinary approach, with chemotherapy being the mainstay of treatment. In this case, the patient was referred to the oncology department and was started on R-CHOP (rituximab, cyclophosphamide, doxorubicin, vincristine, and prednisone) chemotherapy regimen.

The treatment of NHL requires a precise understanding of the disease's biology and its response to therapeutic interventions. Early diagnosis and appropriate treatment are crucial for improving patient outcomes and achieving remission. This case underscores the need for healthcare professionals to consider NHL in the differential diagnosis of persistent tonsillar hypertrophy. Thorough clinical evaluation, imaging studies, and histopathological analysis are essential for accurate diagnosis and appropriate management. The therapeutic approach often involves chemotherapy regimens such as R-CHOP, which is widely used as first-line therapy. Radiotherapy may be considered in cases with localized disease or as adjunctive therapy following chemotherapy. Early recognition and treatment of NHL can significantly impact patient outcomes and improve prognosis.

This case can be linked to two other cases of similar presentation and diagnosis. One described a case of a 72-year-old woman presenting with a sore throat and a smooth, non-tender mass in the left palatine tonsil. Routine laboratory tests were normal, but a computed tomography (CT) scan revealed tonsillar hypertrophy. Histopathological examination confirmed the diagnosis of NHL [20]. Another case involved a 50-year-old man with HIV who presented with symptoms resembling tonsillitis. Further investigation revealed that the underlying cause was NHL, highlighting the importance of considering lymphoma in differential diagnoses, especially in immunocompromised patients [6].

Several risk factors have been associated with the development of non-Hodgkin lymphoma (NHL), particularly in the tonsillar region. Age and gender play a significant role, as tonsillar lymphomas predominantly occur in elderly men, with a peak incidence in the sixth and seventh decades of life [21]. However, younger individuals can also be affected, especially in the presence of other risk factors. Additionally, certain viral infections, particularly the Epstein-Barr virus (EBV), have been implicated in the pathogenesis of NHL. Studies have demonstrated a strong correlation between EBV and lymphoid malignancies, with viral oncogenesis playing a crucial role in NHL development, especially in cases involving Waldeyer's ring. The presence of EBV DNA in tumor tissues of patients with extranodal lymphomas, including those affecting the tonsils, further supports this association [22]. Other environmental and genetic factors may also contribute, but their roles remain less clearly defined.

The prognosis of tonsillar NHL depends on multiple factors, including histological subtype and disease stage at diagnosis. Diffuse large B-cell lymphoma (DLBCL) is the most commonly identified histological subtype in tonsillar NHL. While aggressive, it often presents as a localized disease, which is associated with better treatment outcomes when managed appropriately [21]. Patients diagnosed in the early stages of NHL typically have a favorable prognosis, particularly when the disease is confined to the tonsils without systemic involvement. However, the presence of systemic symptoms, such as fever, night sweats, and unintentional weight loss, indicates a more advanced stage and a higher tumor burden, which can significantly worsen outcomes [20].

## Conclusions

This case emphasizes the importance of recognizing non-Hodgkin lymphoma, specifically follicular lymphoma and DLBCL, as a potential cause of persistent bilateral tonsillar hypertrophy. Thorough clinical evaluation, including imaging and histopathological analysis, is essential for accurate diagnosis. A multidisciplinary approach, with chemotherapy as the cornerstone of treatment, remains critical for achieving favorable outcomes. Early detection and prompt management are pivotal in improving prognosis and ensuring optimal patient care.
